# Minor alterations in the intestinal microbiota composition upon Rotavirus infection do not affect susceptibility to DSS colitis

**DOI:** 10.1038/s41598-021-92796-7

**Published:** 2021-06-29

**Authors:** Kedir Hussen Hamza, Emma Dunér, Isabel Ulmert, Armando Arias, Daniel Sorobetea, Katharina Lahl

**Affiliations:** 1 Immunology Section, Lund University, 221 84 Lund, Sweden; 2 Section for Experimental and Translational Immunology, Department of Health Technology, Technical University of Denmark (DTU), 2800 Kongens Lyngby, Denmark; 3 Centro Regional de Investigaciones Biomédicas (CRIB), Universidad de Castilla-La Mancha (UCLM), 02008 Albacete, Spain; 4 Present Address: Department of Pathobiology, School of Veterinary Medicine, University of Pennsylvania, Philadelphia, PA 19104 USA

**Keywords:** Immunology, Mucosal immunology

## Abstract

Viral triggers at the intestinal mucosa can have multiple global effects on intestinal integrity, causing elevated intestinal barrier strength and relative protection from subsequent inflammatory bowel disease (IBD) induction in various models. As viruses can interfere with the intestinal immune system both directly and indirectly through commensal bacteria, cause-effect relationships are difficult to define. Due to the complexity of putatively causative factors, our understanding of such virus-mediated protection is currently very limited. We here set out to better understand the impact that adult enteric infection with rotavirus (RV) might have on the composition of the intestinal microbiome and on the severity of IBD. We found that RV infection neither induced significant long-lasting microbiota community changes in the small or large intestine nor affected the severity of subsequent dextran sulfate sodium-induced colitis. Hence, adult murine RV infection does not exert lasting effects on intestinal homeostasis.

## Introduction

The intestinal immune system is segregated from the faecal content-containing lumen by a single layer of epithelial cells. How the delicate balance between immune homeostasis in this environment rich in commensal as well as pathogenic microorganisms is controlled is a subject of intense research^1–3^. Inflammatory bowel diseases (IBD), which comprise Crohn’s disease (CD) and ulcerative colitis (UC), are chronic intestinal inflammatory diseases that result from a breakdown of the dynamic equilibrium between the host immune system and environmental factors. Though the precise pathogenesis of IBD is unknown and certainly multifactorial, many studies indicate that dysbiosis, which classically refers to an altered composition of the microbiota, is a driver of disease progression^4–6^. Alterations in viral signatures however also correlate with IBD incidence^7^. The vast majority of viral sequences in the intestines are derived from prophages, which were shown to indirectly affect intestinal health by interfering with microbial diversity, and directly by inducing immune signalling at the intestinal wall^7^. The intestines are however also constantly exposed to chronic as well as acute infections by eukaryotic viruses directly infecting host cells, with context-dependent detrimental as well as beneficial consequences on intestinal health (reviewed in^8^). Broadly applicable solutions for the management of IBD therefore likely require the involvement of the virome as an additional important factor influencing intestinal health.

Viruses and viral products are recognized by host pattern-recognition receptors, which include toll-like receptors (TLRs), retinoic acid inducible gene I (RIG-I)-like receptors (RLRs) or Nucleotide oligomerization domain-like receptors (NLRs)^9–12^. Studies performed in gene-deficient mouse models and mice treated with antiviral cocktails suggest an important role for viral immune signalling in augmenting intestinal barrier strength both at steady-state and in the context of dextran sulfate sodium (DSS)-induced colitis, a model commonly used to mimic acute intestinal inflammation in mice. RIG-I deficient mice spontaneously develop a colitis–like phenotype, and RIG-I, Nalp3 or Mitochondrial antiviral signalling protein (MAVS) deficient mice exhibit susceptibility to DSS-induced colitis with varying levels of severity^11,13–16^. TLR3/TLR7 double-deficient mice mimic antiviral cocktail treated mice in their shared susceptibility to DSS-induced colitis^17^. The combined occurrence of TLR3 and TLR7 genetic variation has also been implicated in human IBD^17^. Norovirus, prominently known as a relevant enteric pathogen causing severe intestinal distress, can protect antibiotics-treated mice from fulminant DSS colitis^18^. Conversely, Norovirus can trigger colitis in genetically predisposed mice, a phenomenon coined “Virus-Plus-Susceptibility Gene interaction”^19^. Together, these findings show that viruses, in addition to their known destructive effects on human health, can be important contributors to the maintenance of intestinal immune homeostasis.

Rotavirus (RV) is a non-enveloped triple-layered segmented double-stranded RNA (dsRNA) virus that primarily infects mature intestinal epithelial cells (IECs). It is the most common cause of severe gastroenteritis and dehydrating diarrhoea in infants and young children worldwide, leading to over 200,000 deaths annually^20^. Adult RV infection is asymptomatic and the triggered response resembles that induced by live-attenuated vaccines^21^. In fact, adult mice challenged with inactivated RV daily for 2 weeks showed attenuated colitis symptoms after DSS administration, suggesting that constant immune stimulation with rotaviral components in the absence of infection might benefit the host^17^. Interestingly, RV vaccination inversely correlates with the incidence of type 1 diabetes (T1D)^22,23^, and it is not clear whether this is due to events following the vaccination, or due to the resulting protection from infection in children^24–26^. The role that RV infection and vaccination play in local and systemic immune imprinting towards foreign and self is thus an important topic of significant impact.

We here set out to better understand the impact that adult RV infection might have on intestinal homeostasis. We found no significant changes in the microbial diversity and richness of infected animals in the small intestine (SI) or large intestine (LI), which differs from neonatal infection models^27–29^, but importantly mirrors findings following neonatal vaccinations^30,31^. In addition, live RV infection did not change disease severity following DSS colitis induction in adult mice. Our findings underpin the resilience of microbial and immune homeostasis at the intestinal wall by showing that temporary RV infection does not per se have long-term consequences on intestinal health in adult wild type mice.

## Materials and methods

### Mice

Female wild type C57BL/6JNR 10-week-old mice were used in the study. The mice were bought from Janvier Labs, Saint Berthevin Cedex, France and were acclimatized for two weeks in the animal facility at Lund University before use. All animal experiments were performed under the animal care and use regulations of the Lund/Malmö Animal Ethics Committee (Malmö—Lunds djurförsöksetiska nämnd) and following the ethical approval for this study (ethical approval number 5.8.18-04525/2017, study leader Katharina Lahl). Experiments were described following the ARRIVE guidelines.

### Rotavirus infection model

The Rotavirus model was performed as previously described^32^. The virulent wild type EC_w_ strain of RV was propagated and collected from suckling mice. The suckling mice were orally gavaged with RV at 3 days of age and sacrificed 2 days after infection to collect clear intestinal homogenates. RV was inoculated orally at a dose of 3 × 10^3^ DD_50_ to infect adult mice. RV antigen shedding was checked in faeces using ELISA. Faecal samples from control and infected mice were collected, weighed, and soaked in PBS with 1% BSA, 1 mM EDTA, 0.05 mg/ml Soybean trypsin inhibitor, 2 mM Phenylmethylsulfonyl floride (PMSF) (Sigma) and 0.025% Sodium-Azide for 2 h at 100 mg/ml. The supernatant was collected after homogenization and centrifugation for 10 min at 14,000 rpm. RV antigen was detected in the supernatant by ELISA using guinea pig anti-RV hyperimmune serum, rabbit anti-RV hyperimmune serum (both kind gifts from the Greenberg laboratory), goat anti-rabbit IgG-HRP (Millipore) and TMB Substrate Reagent Set (RUO, 555214, BD Biosciences) as previously described^33^.

### Dextran sulphate sodium (DSS) induced colitis

The DSS model was performed as previously described with some modifications^17^. 1–3% (w/v) of DSS (MW: 36,000–50,000; MP Biomedicals, CA) was dissolved in sterilized drinking water (pH ~ 7) and given ad libitum for 8 or 9 days to induce colitis, followed by 1 day of water. Mice were weighed every day to determine percentage weight changes. Mice were monitored clinically for rectal bleeding and diarrhoea. For RV groups, DSS was introduced to mice that received RV 6 days or 14 days prior to DSS. Clinical scoring of a disease activity index (DAI) was also performed as previously described^17^.

### Intestinal content sampling for bacterial extraction

Intestinal contents were collected from the small and large intestine using a filtered Phosphate-buffered saline (PBS) with protease inhibitor (Sigma). The mucosal layer was scraped to include the communities closer to the mucosal wall. The samples were vigorously vortexed and centrifuged to collect the bacteria. All samples were stored at − 80 °C until further processing. Total Bacterial DNA was extracted using QIAamp PowerFecal DNA Kit (MO BIO, QIAGEN) following the manufacturer’s instructions.

### 16S rRNA gene sequencing and analysis

Samples were prepared using 16S Metagenomics Sequencing Library Preparation protocol (#15044223) for sequencing the variable V3 and V4 regions of the 16S rRNA gene. In short, PCR amplification was performed spanning the V3-V4 region and Illumina sequencing adapters and dual-index barcodes to the amplicon target were added. PCR clean-up was done using AMPure XP beads after amplicon PCR and Indexing PCR. Libraries were validated using Agilent 2100 Bioanalyzer and quantified using Picogreen. Subsequently, samples were normalized, pooled, and sequenced using Illumina Miseq platform at the Department of Biology, Lund University DNA Sequencing Facility. Samples were sequenced using 300-bp paired-end sequencing and generate high-quality, full-length reads of the V3 and V4 region of 16S rRNA gene. Illumina BaseSpace obtained FASTQ sequence data were imported into Quantitative Insights Into Microbial Ecology 2 (QIIME2) 2020.2^34^ in the Casava 1.8 paired-end demultiplexed FASTQ format. Sample metadata was validated using Keemei^35^. Sequence quality control and amplicon sequence variant (ASV) picking were carried out using DADA2^36^. Alpha rarefaction curves were plotted using QIIME 2 alpha-rarefaction and were used to set an appropriate subsampling depth for each comparison. Samples were rarefied using QIIME 2 feature-table rarefy. Samples with a read depth lower than the relevant subsampling depth were excluded from the analysis. ASVs were assigned with taxonomic annotations using a naïve Bayes fitted classifier trained on the Greengenes 13_8 99% OTUs^37^. Relative abundance tables were calculated using QIIME 2 feature-table summarize-taxa. We calculated various measures of alpha diversity using the methods implemented in the MicrobiomeAnalyst^38^. Ordination plots were also calculated from Bray–Curtis distance matrix in NMDS coordination method using MicrobiomeAnalyst^38^. Sequencing data is deposited in the European Nucleotide Archive (ENA) at EMBL-EBI under accession number PRJEB44696 (https://www.ebi.ac.uk/ena/browser/view/PRJEB44696).

### Cell isolation and flow cytometry

After removing the epithelial cells from the colon lamina propria (cLP), single cell suspensions were prepared by enzymatic digestion, as previously described^39^. In short, the colon was opened longitudinally and cut into 1 cm pieces. Epithelial cells and mucus were removed by incubating tissue in HBSS supplemented with FCS (2%) and EDTA (2 mM) 3 times each for 15 min at 37 °C with continual shaking at 450 rpm. The remaining tissue pieces were incubated in R10 complete medium (RPMI 1640 supplemented with FCS (10%), HEPES (10 mM), sodium pyruvate (1 mM), penicillin (100 U/ml), streptomycin (100 μg/ml), gentamicin (50 μg/ml) and 2-mercaptoethanol (50μM)) containing DNase I (12.5 μg/ml) and Liberase (0.3 U/ml) for 45 min at 37 °C. Subsequently, the resulting cell suspensions were filtered through 100-μm cell strainers (Fisher Scientific) for further analysis. Flow cytometry was performed according to standard procedures. Non-specific binding was blocked with rat serum (Sigma) and rat anti-mouse CD16/32 Fc block (2.4G2, BD Biosciences) for 20 min at 4 °C. The following antibodies were used to stain the cells; BioLegend: anti-CD45.2 (104), NK1.1 (PK136), CD3 (145-2C11), CD19 (MB19-1) and MHC class II (MHCII; M5/114.15.2). BD Biosciences: anti-CD64 (× 54–5/7.1), Ly-6C (AL-21), Ly-6G (1A8), Siglec-F (E50-2440), CD103 (M290) and PE-CF594 streptavidin. From eBioscience: anti-CD11c (N418) and CD11b (M1/70). Dead cells were identified using propidium iodide (PI). Flow cytometry data was acquired on an LSR II (BD Biosciences) and analysed using FlowJo software v10.7.1 (Tree Star).

### Quantitative real-time PCR

Snap frozen tissue was disrupted and homogenized using a TisssueLyser II (Qiagen). Total RNA was isolated using RNeasy Mini Kit (Qiagen), and genomic DNA was degraded using a DNase treatment step according to the manufacturer’s protocol. Complementary DNA was generated using iScriptTM cDNA Synthesis Kit (Bio-Rad). Quantitative real-time PCR was performed on a CFX96TM Real-Time PCR Detection System (Bio-Rad) using KAPA SYBR FAST qPCR Master Mix (2X) Universal (Kapa Biosystems). QuantiTect Probe PCR kit (Qiagen) was used for the probe-based qPCR for the detection of RV VP4. All samples were run in duplicates and expression levels of genes were normalized to the mean of Gapdh. Primer sequences (IDT) are as follows: Isg15 forward 5′-gag cta gag cct gca gca at-3′ and reverse 5′-ttc tgg gca atc tgc ttc tt-3′; Mx1 forward 5′-tct gag gag agc cag acg at-3′ and reverse 5′-act ctg gtc ccc aat gac ag-3′; Oasl2 forward 5′-gga tgc ctg gga gag aat cg-3′ and reverse 5′-tcg cct gct ctt cga aac tg-3′; Gapdh forward 5′-tgc acc acc aac tgc tta gc-3′ and reverse 5′-ggc atg gac tgt ggt cat gag-3′; RV VP4 (EC strain) forward 5′-agt ggc taa gca cac gga cta-3′ and reverse 5′-cgg agt gtg cgg tca ga-3′ and probe 5′-6-FAM-cga ctg cag gtc agg cat act acg c/TAMRA-3′; HPRT forward 5′-ggc cag act ttg ttg gat ttg-3′ and reverse 5′-cgc tca tct tag gct ttg tat ttg-3′ and probe 5′-6-FAM-cca gac aag/ZEN/ttt gtt gtt gga tat gcc c/3IAbkFQ-3′. Negative-control samples contained no RNA and undetectable values were calculated based on the highest possible Cq + 1 (= 46 cycles).

### Statistical analysis

GraphPad Prism software was used for statistical analysis. Significant differences among multiple groups were analysed by one-way ANOVA followed by Tukey post hoc test (*P < 0.05; **P < 0.01; ***P < 0.001).

## Results

### Rotavirus infection does not cause significant restructuring of the murine intestinal microbiota

**Figure 1:**
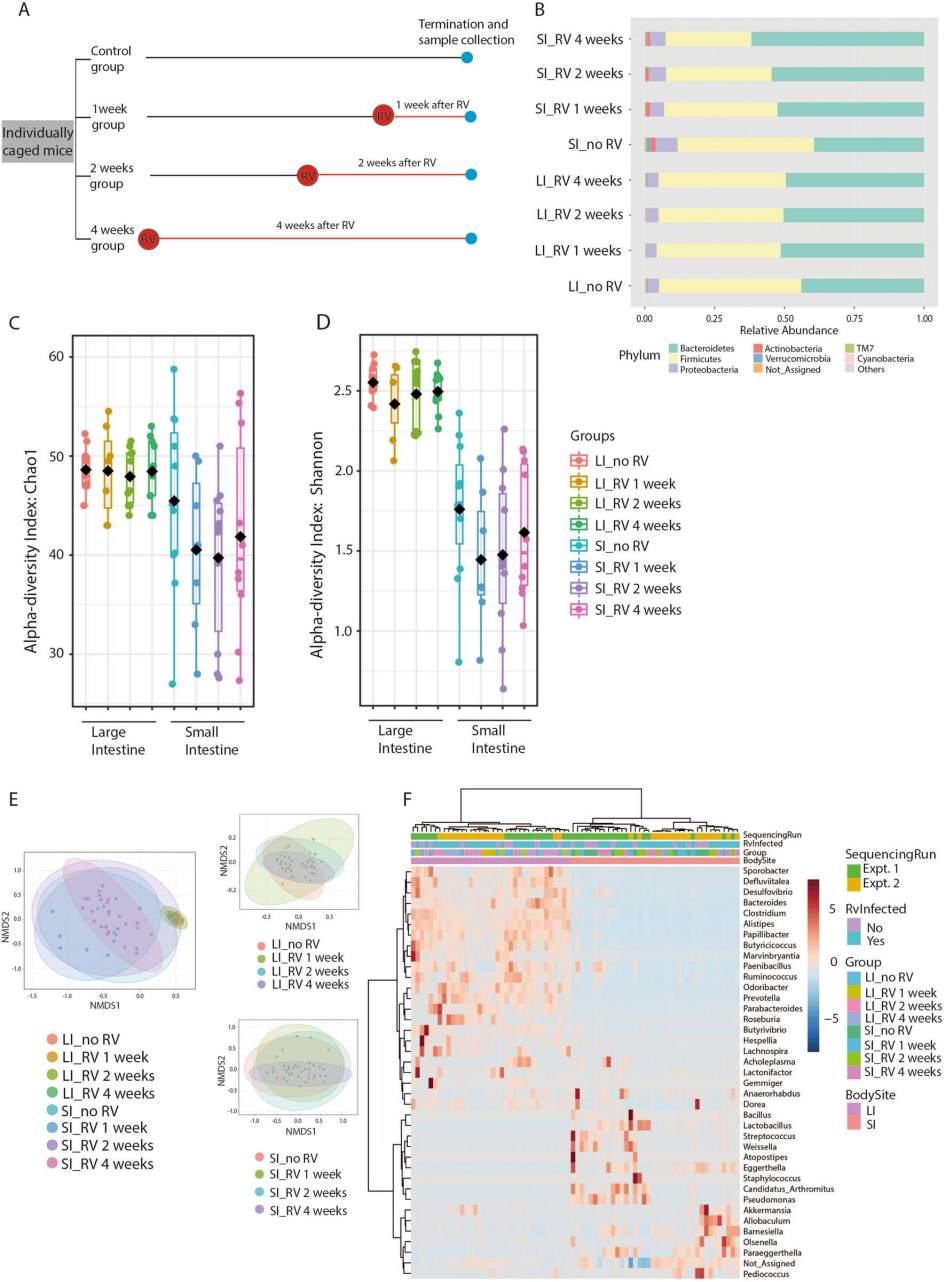
Dynamic of the intestinal microbiota composition upon RV infection. (**A**) Image illustrating the study design. (**B**) Taxonomic composition of community using Stacked Bar plot. Relative abundance of the top 8 Phylum in SI and LI in merged samples to groups (SI and LI; uninfected and 1, 2, and 4 weeks post infection for each niche). (**C**,**D**) Alpha diversity at the bacterial Genus level represented by Chao1 index (**C**) and Shannon index (**D**) represented as boxplot. Each boxplot represents the diversity distribution of the group. (**E**) Beta diversity at the bacterial Genus level determined by Nonmetric Multidimensional Scaling (NMDS). (**F**) Heat-map depicting the abundance of major bacterial taxa at the Genus level. Results are from a total of 76 intestinal samples (38 from SI and 38 from LI) from two independent experiments.

Murine RV productively infects adult mice without causing pathology^21^. Viral titres of the ECw strain in adult mice peak at 4–5 days and the virus is commonly cleared around day 7 post infection^32,40^. To assess whether infection of adult mice with RV influenced the composition of the microbiota, we performed 16S rRNA-targeted amplicon Illumina sequencing on SI and LI samples from individually caged mock and infected female mice at 1, 2 and 4 weeks post infection (Fig. 1A). As expected, fecal shedding of RV peaked at 4 days post infection (Suppl. Fig. S1A) and virus was also detectable by qPCR on virus protein 4 (VP4) at this time point in samples of adult ileum (Suppl. Fig. S1B). We collected a total of 76 intestinal samples (38 from SI and 38 from LI) from two separate individually sequenced experiments.

We detected a total of 173 features following Amplicon sequence variants (ASV) Greengenes database annotation using QIIME2^34,38^. Quality filtering and rarefying to 37,729 high-quality paired-end sequences resulted in 38 SI and 36 LI samples. The individual library size for each sample is shown in supplementary figure S2A. We explored the rarefaction and observed sufficient sampling depth for community analysis (Suppl. Fig. S2B). For downstream statistical analysis, we removed features with less than 4 counts and a prevalence of less than 10% in all samples, excluding a total of 28 low abundance features. In addition, a total of 9 low variance features were removed based on standard deviation. After data filtering, 72 features remained for the subsequent analysis.

We analysed the relative abundance of different bacterial phyla in both SI and LI niches of the control and RV infected groups. Each niche showed its own community structure (Fig. 1B). The core microbiota detected across all samples included Bacteriodetes, Firmicutes, Proteobacteria and Actinobacteria (Suppl. Fig. S3A). As expected, Bacteriodetes and Firmicutes, followed by Proteobacteria were the dominant phyla in both the SI and the LI^41,42^, and this was not changed by RV infection (Fig. 1B). The relative abundance of Actinobacteria, a phylum known to contribute to the microbiota across the entire length of the gastro-intestinal tract^43^, was overall visibly higher in the SI (Fig. 1B). Its significance and effect size were confirmed using Linear Discriminant Analysis (LDA) Effect Size (LEfSe) (Suppl. Fig. S3B).

α-diversity analysis of both the SI and LI microbiota showed that both taxonomic richness (Chao1 *P* value: 0.013148; [ANOVA] F-value: 2.7789) and diversity (Shannon *P* value: 2.7697e-13; [ANOVA] F-value: 18.287) were significantly different between niches (Fig. 1C,D). Overall, the α-diversity within LI samples showed much less variation within groups and was not affected by RV infection. In contrast, we detected a high variation between individual SI samples within groups and a non-significant trend towards decreased richness and diversity in the first 2 weeks following RV infection and returning towards baseline by week 4 (Fig. 1C,D). Bray–Curtis dissimilarity index analysis of β-diversity confirmed that the microbiota between niches was highly dissimilar regardless of RV infection ([PERMANOVA] F-value: 10.961; R-squared: 0.53758; *P* value < 0.001

[NMDS] Stress = 0.077533). This was obvious despite the much higher population variance within the SI samples ([PERMDISP] F-value: 4.6803; *P* value: 0.00026359 [NMDS] Stress = 0.077533) (Fig. 1E). On the other hand, β-diversity NMDS and PERMANOVA analysis of Bray–Curtis dissimilarity index upon RV infection revealed no significant difference between experimental groups in either SI (PERMANOVA F-value: 1.1207; R-squared: 0.08999; *P* value < 0.331 [NMDS] Stress = 0.12498) or LI niches (PERMANOVA F-value: 1.0177; R-squared: 0.087098; *P* value < 0.414 [NMDS] Stress = 0.16872) (Fig. 1E), meaning that RV infection did not significantly affected microbial constitution.

For a more granular view of potentially induced changes of the microbial communities upon RV infection, we performed a heatmap clustering of all samples at the genus level (Fig. 1F). The sample clustering was primarily driven by the niche (SI vs LI), followed by batch effects of our first and second sequencing run. RV infection did not cause dominant clustering, but there was a trend towards non-random distribution of samples between different infection groups within batches (Fig. 1F).

**Figure 2:**
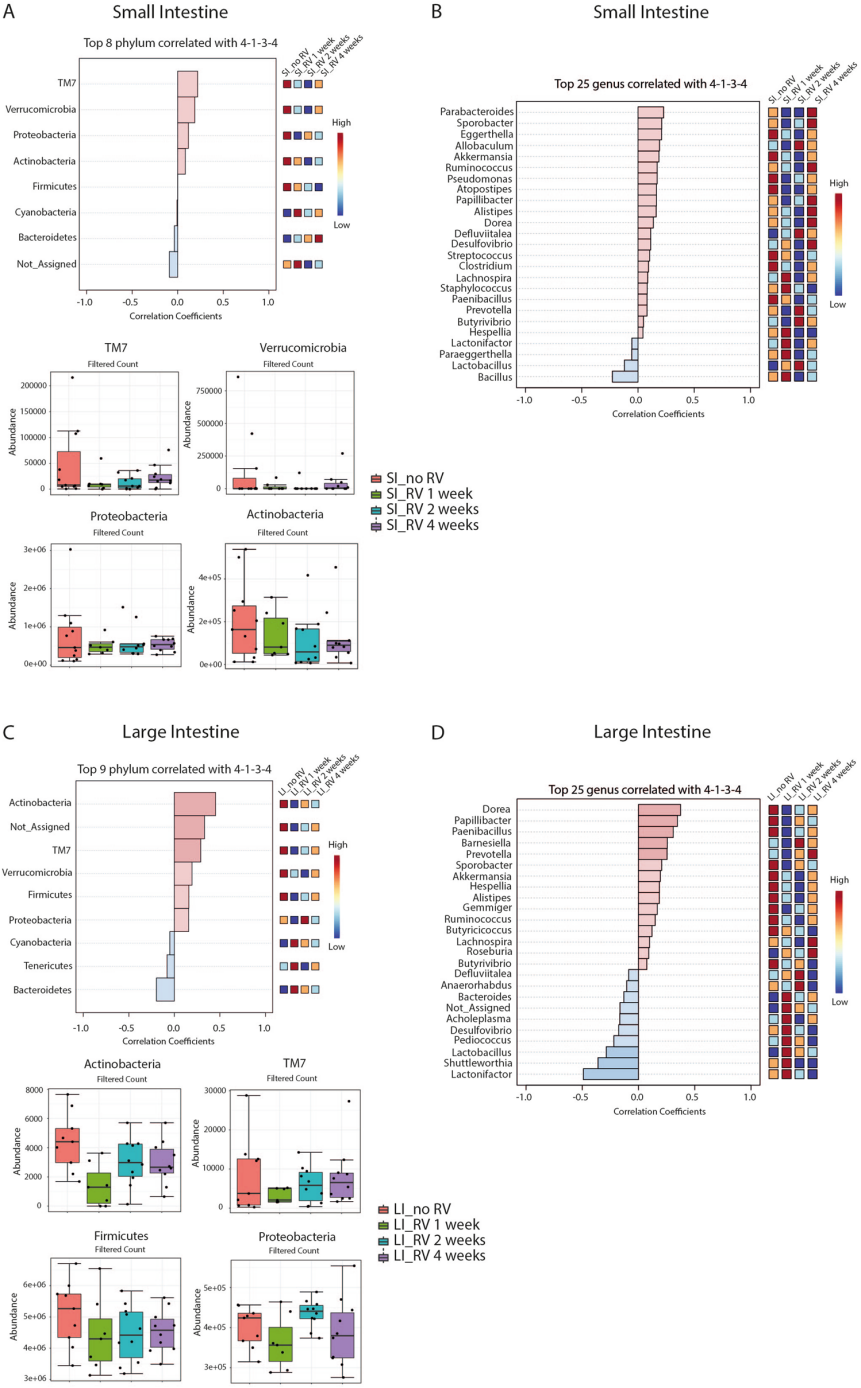
Important features identified by Pattern search using correlation analysis. The features are ranked by their correlation, and the light blue bars represent negative correlations, while light red bars represent positive correlations. To the right is a mini-heatmap showing whether the abundance of that features is higher (red) or lower (blue) in each group. (**A**) Important features from the SI niche at the Phylum level. (**B**) Important features from the SI niche at the Genus level. (**C**) Important features from the LI niche at the Phylum level. (**D**) Important features from the LI niche at the Genus level. Results are from a total of 76 intestinal samples (38 from SI and 38 from LI) from two independent experiments.

Individual differences in specific features might affect intestinal health without causing significant shifts in overall diversity and clustering. We therefore performed linear discriminant analysis (LDA) of effect size (LEfSe). Again, we could not identify significant changes upon RV infection in SI or LI (Supplementary Table S1A&B). We next asked whether the trend observed in the α-diversity of the SI (Fig. 1D) was driven by specific taxa, or whether it reflected a rather general pattern in the dataset. For this, we chose a pre-defined pattern of 4-1-3-4 to fit the α-diversity (Shannon index) trend (decreased diversity at 1 and 2 weeks after RV infection). At the phylum level TM7, Verrucobacteria, Proteobacteria and Actinobacteria showed a positive correlation with this non-significant trend, albeit with a very low correlation coefficient (0.22 for TM7) (Fig. 2A). Hence, no specific phyla were significantly correlated with this predominant pattern. This was also true when analysing on the genus level, where Barnesiella, Parabacteroides, Sporobacter, Eggerthelia, Allobaculum, Akkermansia and Ruminococcus were the top 7 genera correlated with the chosen pattern (Fig. 2B and Suppl. Fig. S4A). Despite an even smaller difference in α-diversity between the groups of LI samples, some phyla at this site, namely Actinobacteria and TM7, correlated somewhat better with the 4–1-3–4 pattern than they did in the SI (Fig. 2C), even though these phyla are relatively more abundant in the SI (Fig. 2B). On the genus level, this was reflected by several positive correlations, of which Dorea, Papillibacter, Paenibacillus, Barnesiella and Prevotella were dominant (Fig. 2D and Suppl. Fig. S4B).

Taken together, RV infection did not induce significant long-lasting microbiota community changes in the SI or LI.

### Prior exposure to RV infection does not alter the susceptibility to DSS-induced colitis in C57/B6 adult female mice

**Figure 3:**
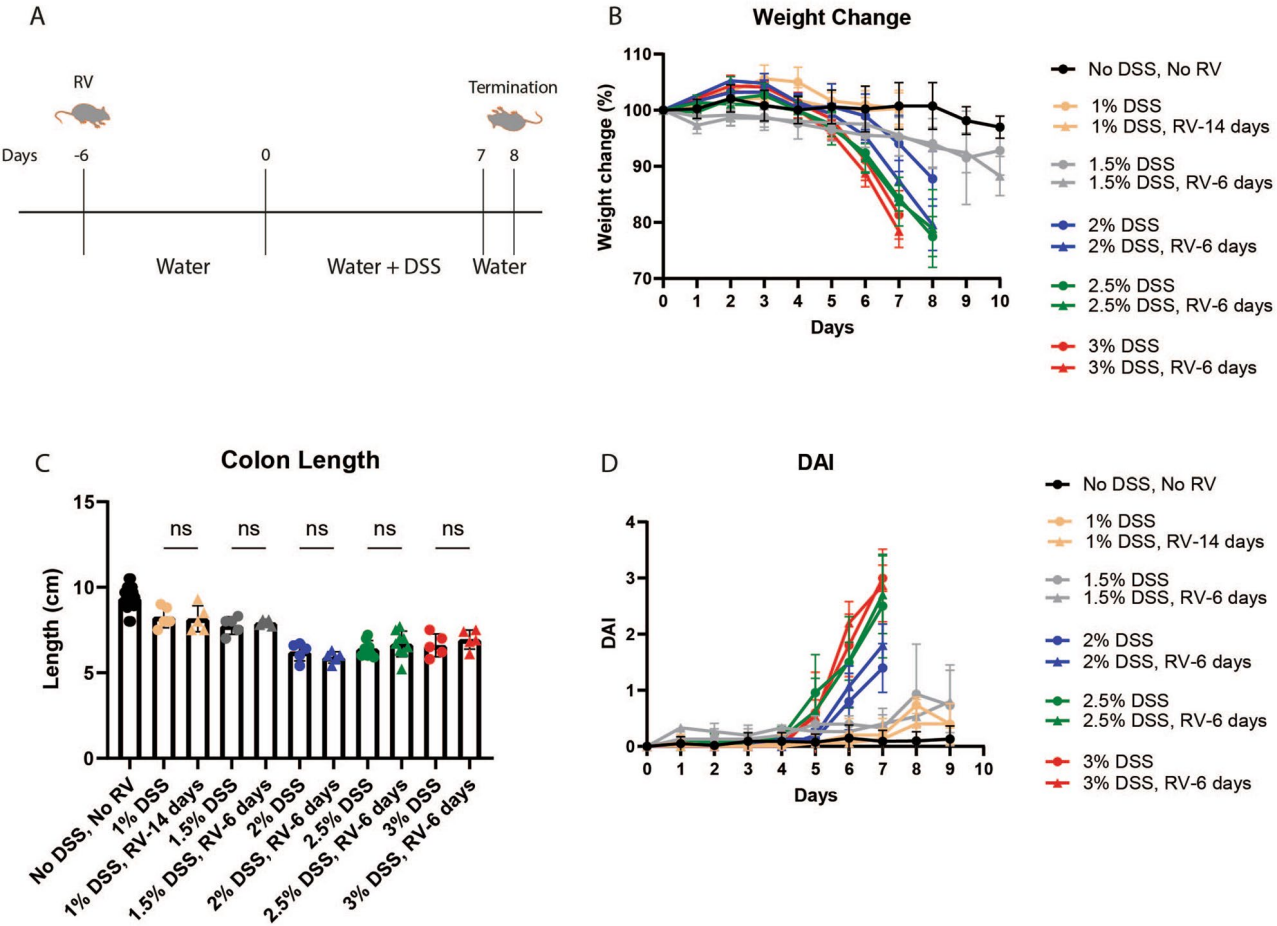
Effect of prior RV infection on clinical symptoms of acute DSS-induced colitis. (**A**). Schematic representation of the study timeline. Wild type C57BL/6NRj mice were categorized into 3 groups (control, only DSS and DSS with prior RV infected groups). Administered 1% to 3% DSS in drinking water for 7 to 9 days and followed by 1 day of normal drinking water. Mice were monitored daily. (**B**) Body weight was measured daily and means ± SD of percent body weight were plotted. (**C**) Colon length was measured as an indirect marker of inflammation. (**D**) Disease activity index was calculated as follows: DAI = (weight loss + stool consistency + gross bleeding)/3). Results are shown as mean ± SD. A total of 80 mice included from four independent experiments. All 2%, 2.5% and 3% DSS where administered for 7 days followed by 1 day with water (DSS started 6 days post RV infection). 1% DSS was administered for 8 days followed by 1 day with water (DSS started 14 days post RV infection) and 1.5% DSS was administered for 9 days followed by 1 day with water (DSS started 6 days post RV infection).

We next sought to understand the role of RV infection in alleviating gut inflammation, which could be either driven by the subtle differences that we observed in the microbiome one and two weeks after infection, or directly by viral sensing through the immune system. To this end, we induced acute DSS colitis 6 or 14 days after RV infection (Fig. 3A). We used a large range of DSS concentrations, as a potential positive impact of viral signalling could be hidden in case of fulminant inflammation.

DSS administration caused severe weight loss (> 10%) starting on day 5 for 3% and 2.5% DSS, and on day 6 for 2% DSS. Mice receiving 1.5% DSS also lost weight, but to a much lower extent than those mice treated with higher concentrations. 1% DSS in the drinking water did not induce weight loss in our cohort. Regardless of the DSS concentration, mice previously infected with RV lost weight to the same extent as their matched controls (Fig. 3B). No gross variation in water consumption was noted, which implies that DSS uptake was similar between infected and uninfected groups. Weight loss was closely correlated with a decrease in colon length (Fig. 3C) and the overall disease activity index (DAI), which is a merged score derived from weight loss, stool consistency and rectal bleeding parameters (Fig. 3D). Neither the DAI nor any individual parameter differed between RV-infected and uninfected DSS-treated groups. As expected in the adult infection model, RV infection did not cause any weight loss per se (Suppl. Fig. S5). Hence, RV infection did not ameliorate inflammatory bowel disease symptoms as assessed by the DSS model of colitis.

**Figure 4:**
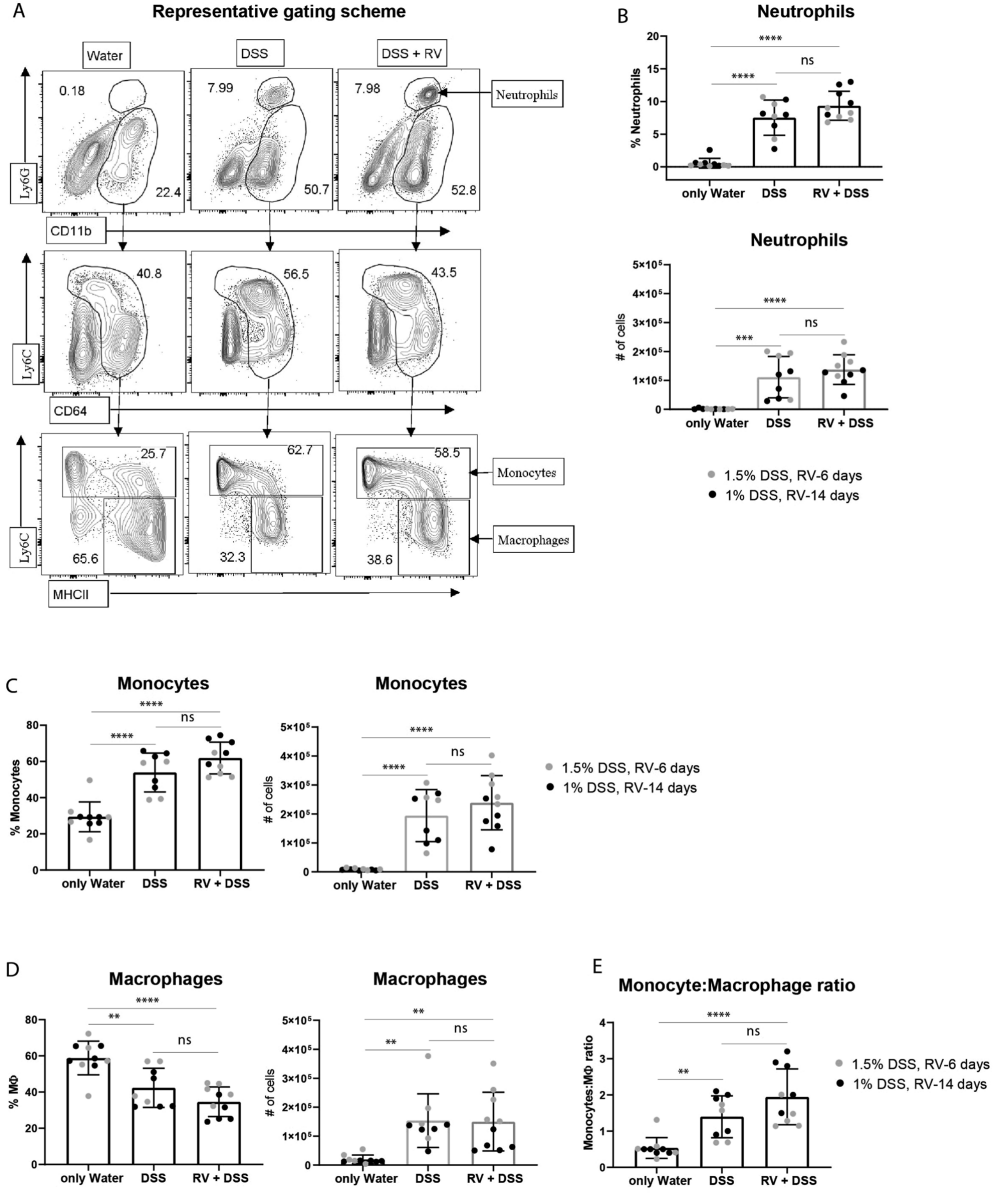
Effects of DSS-induced colitis on the recruitment of monocytes to the colon. Monocyte infiltration into the colon was assessed after induction of inflammation by DSS. (**A**) Representative gating scheme from 1% DSS (RV-14 days experiment, 9 days post DSS onset). Representative expression of CD11b and Ly6G from live-gated CD45^+^ colonic cells (upper panels), expression of CD64 and Ly6C from CD11b^+^Ly6G^-^ colonic cells (middle panels), and expression of MHCII and Ly6C from CD11b^+^Ly6G^-^CD64^+^Ly6C^hi/lo^ (lower panels) in control, only DSS and DSS with prior RV infected groups. Neutrophils are shown as CD11b^+^Ly6G^hi^. Monocytes are defined as CD11b^+^Ly6G^-^CD64^+^Ly6C^hi^MHCII^+/-^. Macrophages are defined as CD11b^+^Ly6G^-^CD64^+^Ly6C^lo^MHCII^+^. (**B**) Frequency and absolute numbers of neutrophils in the control, DSS or DSS + RV groups. (**C**) Frequency and absolute numbers of monocytes in the control, DSS or DSS + RV groups. (**D**) Frequency and absolute numbers of macrophages in the control, DSS or DSS + RV groups. (**E**) Ratios of Monocyte: Macrophage in the control, DSS or DSS + RV groups. Results are shown as mean ± SD and are derived from two independent experiments with 4–5 mice per group. All data points indicate individual mice. Black symbols indicate data from the experiment in which 1% DSS was administered for 8 days followed by 1 day of water (DSS started 14 days post RV infection), grey symbols indicate data from the experiment in which 1.5% DSS was administered for 9 days followed by 1 day with water (DSS started 6 days post RV infection). (*P < 0.05, **P < 0.01, ***P < 0.001, ****P < 0.0001).

Putative protective effects of prior RV infection on colitis severity might be too subtle for detection by weight loss or DAI. We, therefore, assessed the extent of infiltration of inflammatory immune cell subsets into the colon. We detected an enhanced CD11b^+^Ly6G^hi^ neutrophil infiltration into the colon even at very low DSS concentrations (1% and 1.5%) (Fig. 4A,B). Again, this was not altered by previous RV infection (6 days prior for 1.5% DSS and 14 days prior for 1% DSS) (Fig. 4B).

Under healthy conditions, macrophages dominate the mononuclear phagocyte composition in the murine colon while inflammatory monocytes are recruited upon inflammation^44^. RV infection alone did not cause changes in colonic macrophage and monocyte numbers 6 days post RV infection (Suppl. Fig. S6A), which is consistent with an absence of a significant type I IFN signature in adult intestine at this timepoint (Suppl. Fig. S6B). DSS-induced inflammation triggered monocytes recruitment alongside a reduction of the proportion in macrophages compared to steady-state and this was again unchanged by 6 or 14 days prior RV infection (Fig. 4C–E). Taken together, RV infected C57BL/6JNR adult mice were equally susceptible to DSS-induced colitis as uninfected mice, showing that prior infection with live RV does not ameliorate DSS-induced colitis.

## Discussion

Inflammatory bowel diseases are associated with both viral^7,45^ and bacterial dysbiosis (reviewed in^46^). The complexity of trans-kingdom interactions in the bowel is immense, and cause/effect relationships are extremely difficult to disentangle. Commensal bacteria have been shown to increase enteric viral infectivity through direct and indirect mechanisms^47^, but the effects of enteric viral infections on the intestinal microbiota are less clear. We here demonstrate that temporary, asymptomatic, infection of adult mice with RV does not alter disease severity in a model of acute colitis. This is consistent with the lack of significant changes in the microbial community of both the SI or LI.

Eukaryotic viruses may directly influence the severity of inflammatory bowel diseases, which depending on the viral trigger and host genetics can have both disease-ameliorating as well as disease-exaggerating effects. Depletion of the virome by the administration of virostatica aggravates DSS-colitis^17^, an effect mirrored in mice lacking major nucleic-acid-sensing pathways, such as TLR3/7^17^, RIG-I/MAVS^13,16,48^ and STING^48^. This highly suggests that canonical steady-state sensing of the enteric virome is crucial for intestinal resilience, a finding reminiscent of the role of the indigenous microbiota, depletion of which equally increases susceptibility to intestinal injury caused by the absence of TLR-mediated signaling^49^.

A previous study described that RIG-I-deficiency aggravated radiation-induced intestinal barrier damage^48^. The authors tested whether this was due to community shifts in the microbiome by analyzing co-housed RIG-I-deficient and -sufficient littermates and found no difference^48^. As mice are coprophagic, differences can however be masked in this experimental setup. Treatment of mice with an antiviral cocktail was shown to significantly alter the microbiome in a study for which information on housing conditions was not given^17^. We here decided to analyze single-housed animals, with the caveat that the variance between commensal communities, particularly of those inhabiting the SI, was too large to allow for analysis of minor differences. Higher variance in SI versus LI microbial diversity between individuals was previously described in mice^50^, pigs^51^ and humans^52^ and is probably at least in part due to overall lower colonization at this site. Only two genera (parabacterioides and ruminococci) of those showing a trend of temporary alteration upon RV infection in our study were significantly altered in mice upon treatment with antiviral cocktail^17^: Whilst negatively correlating with RV infection, these genera were more abundant before antiviral cocktail treatment, suggesting that RV infection cannot amplify steady-state effects on the microbiome imprinted by viral signaling. The absence of major lasting alterations of the microbiome induced by murine RV infection is consistent with a previous study describing a very early (day 1 post infection) increase in mucus metabolizing strains and a return to baseline microbiota by day 3 in neonatal mice^27^. Long-term effects were not assessed in this study.

Interestingly, infection with murine norovirus can rescue antibiotics-treated mice from severe disease in the acute DSS colitis model, showing that specific viruses can cover some of the protective effects classically assigned to the microbiome^18^. This protection depended on intact type I IFN signaling on epithelial cells, which was required for monocyte recruitment^53^. CCR2-deficient animals were not protected from severe colitis by Norovirus infection^53^. We here used monocytic influx as a marker positively associated with DSS colitis severity^44,54^ and report no difference in RV infected versus untreated wild type mice. Importantly, RV did not significantly increase basal levels of monocytes in the intestine (Suppl. Fig. S6A), which is either reflecting the difference between the two viruses used, or a difference based on the lack of antibiotic treatment in our model.

In IL-10-deficient mice, murine norovirus infection causes increased barrier permeability, leading to microbiota-dependent mucosal inflammation despite an increase of interferon stimulated genes in susceptible mice^55^. Deficiency in the autophagy gene Atg16l1 in combination with the persistent norovirus strain CR6 led to a significantly pronounced Crohn’s disease phenotype in the murine model of DSS-colitis, a phenomenon referred to as “Virus-Plus-Susceptibility Gene interaction”^19^. Again, colitis induction depended on the presence of microbiota and TNFα/IFNγ signaling. Importantly, the acute strain CW3 did not exacerbate DSS-colitis in Atg16l1-deficient mice, showing that described effects on colitis-pathology can be highly strain-specific^19^. Whether or not RV can exert protective effects in genetically modified mice more susceptible to colitis remains to be determined.

We here induced DSS colitis 6 or 14 days after adult RV infection to test whether RV imprinted durable changes within the microbiota or within the intestinal immune system that might have long-term consequences on intestinal health. Viral clearance in adult mice is usually completed by day 6. Importantly, 1% DSS administration did not alter the viral shedding curve if administered in parallel in a previous study^56^ and RV infection of adult mice is immunogenic but asymptomatic, largely reflecting the response to a vaccine^21^. Our data hence suggest that adult RV infection or vaccination does not impact the microbial community in the intestine and does not influence inflammatory bowel disease severity in the wild type setting. Whether or not symptomatic infection with RV in children influences intestinal health, as has been suggested for Norovirus infection, remains to be investigated.

Our findings seemingly contrast with a previous study reporting that continuous feeding of UV-inactivated RV during DSS treatment ameliorated colitis symptoms^57^. It is possible that the constant triggering of the type I IFN pathway indeed has positive effects on intestinal health. Live RV efficiently inhibits type I IFN signalling through several pathways which might contribute to the absence of positive effects on intestinal health in our study. Indeed, no type I IFN signature was detectable at day 6 post RV infection, the day of DSS treatment onset (Suppl. Fig. S6B).

Due to the multidimensionality of interactions between host, microbiota and virome, identifying major players in sustaining intestinal health requires reductionist approaches in well-controlled settings. Using several readouts, we here show that acute infection of adult mice with homologous RV does not generally impact on intestinal homeostasis and accordingly does not change the disease course of acute DSS colitis. Due to the high abundance of RV infections and existing vaccination programs, together with an increased awareness of the secondary effects of viral infections on host health, our study is an important contribution to our understanding of this highly complex interactome.

## Supplementary Information

Supplementary Figures.

Supplementary Tables.
