# Supplementary material for: Minor alterations in the intestinal microbiota composition upon Rotavirus infection do not affect susceptibility to DSS colitis
Source: Sci Rep. 2021 Jun 29;11:13485. doi: 10.1038/s41598-021-92796-7 (PMC8242028; doi:10.1038/s41598-021-92796-7)
Supplement: Supplementary file 1 — Supplementary Figures. [file 41598_2021_92796_MOESM1_ESM.docx]

**Supplementary File**

**Minor alterations in the intestinal microbiota composition upon Rotavirus infection do not affect susceptibility to DSS colitis**

Kedir Hussen Hamza^1^, Emma Dunér^1^, Isabel Ulmert^2^, Armando Arias^3^, Daniel Sorobetea^1,4^, Katharina Lahl*^1,2^


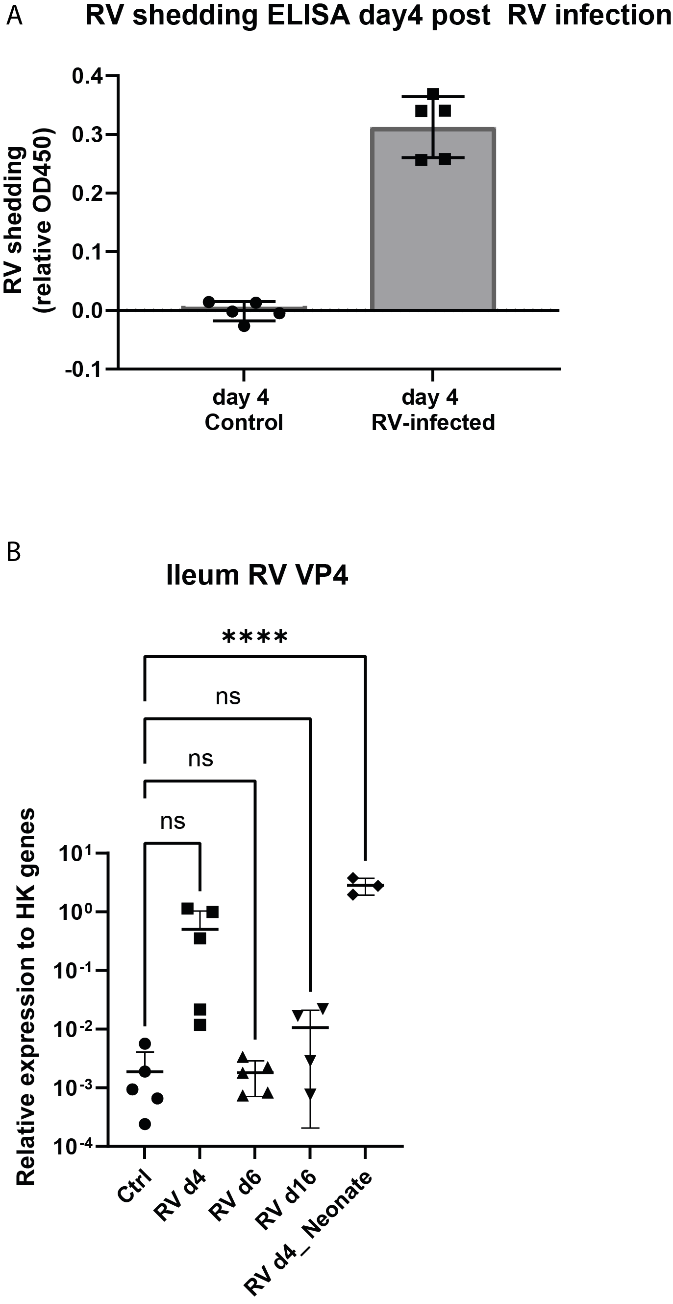


**Supplementary figure 1: RV infection of adult mice.**

(A) Shedding ELISA indicating the shedding of RV antigens in the faeces as measured by ELISA (Day 4 post infection). Data is from 1 experiment with 5 mice per group.

(B) RT-qPCR measuring viral protein (VP) 4 mRNA within ileal tissue from 1 experiment. Samples were taken from 4-5 mice per group at indicated time points after infection. Day 4 neonatal samples were included as reference. Results are shown as mean ± SD. ( ****P < 0.0001)

**
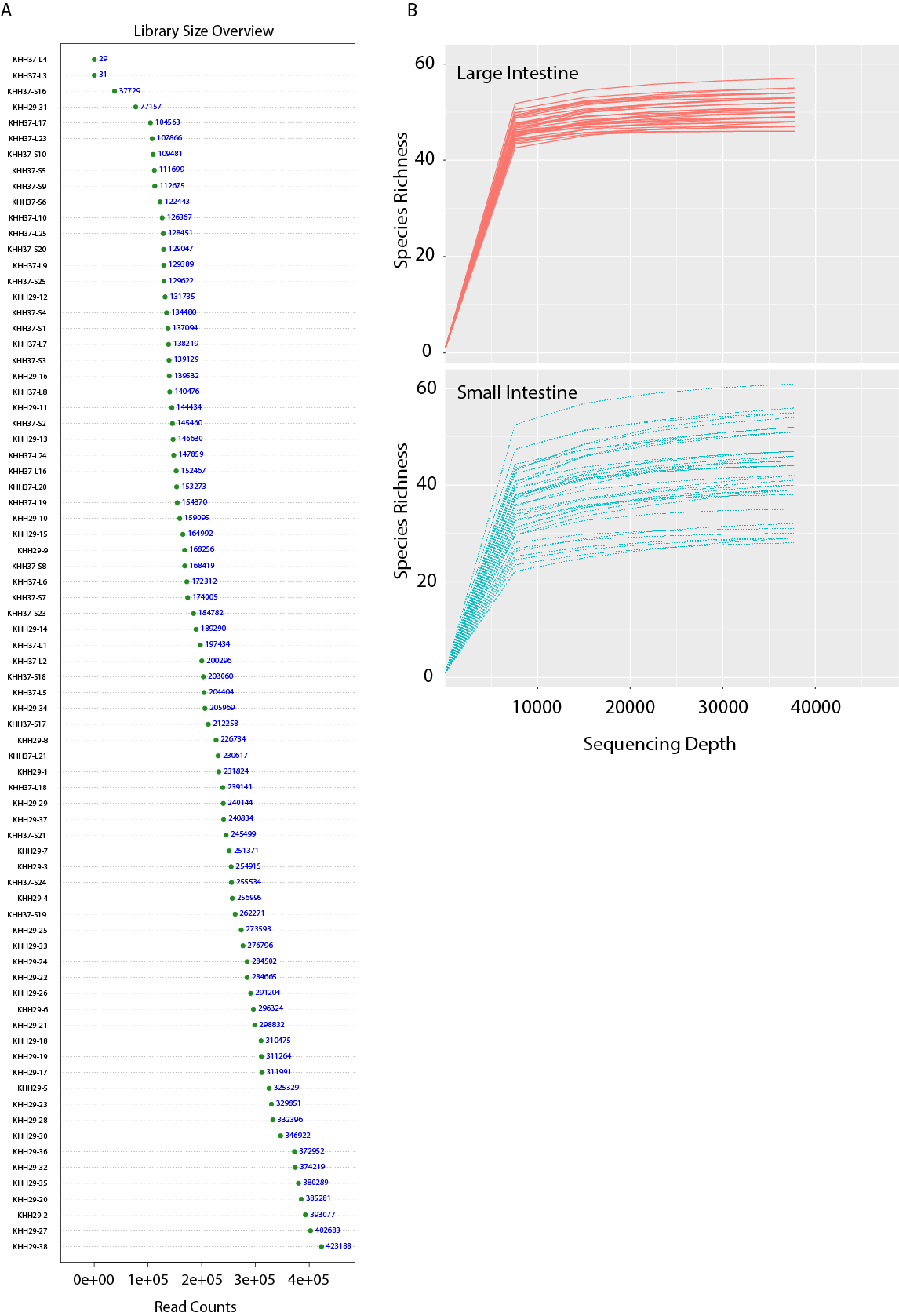
**

**Supplementary figure 2: Microbial data quality check**

(A) Library size for inspection of each sample. Individual read counts of all 76 samples are shown.

(B) Rarefaction curve using filtered dataset for both small and large intestine samples.

**
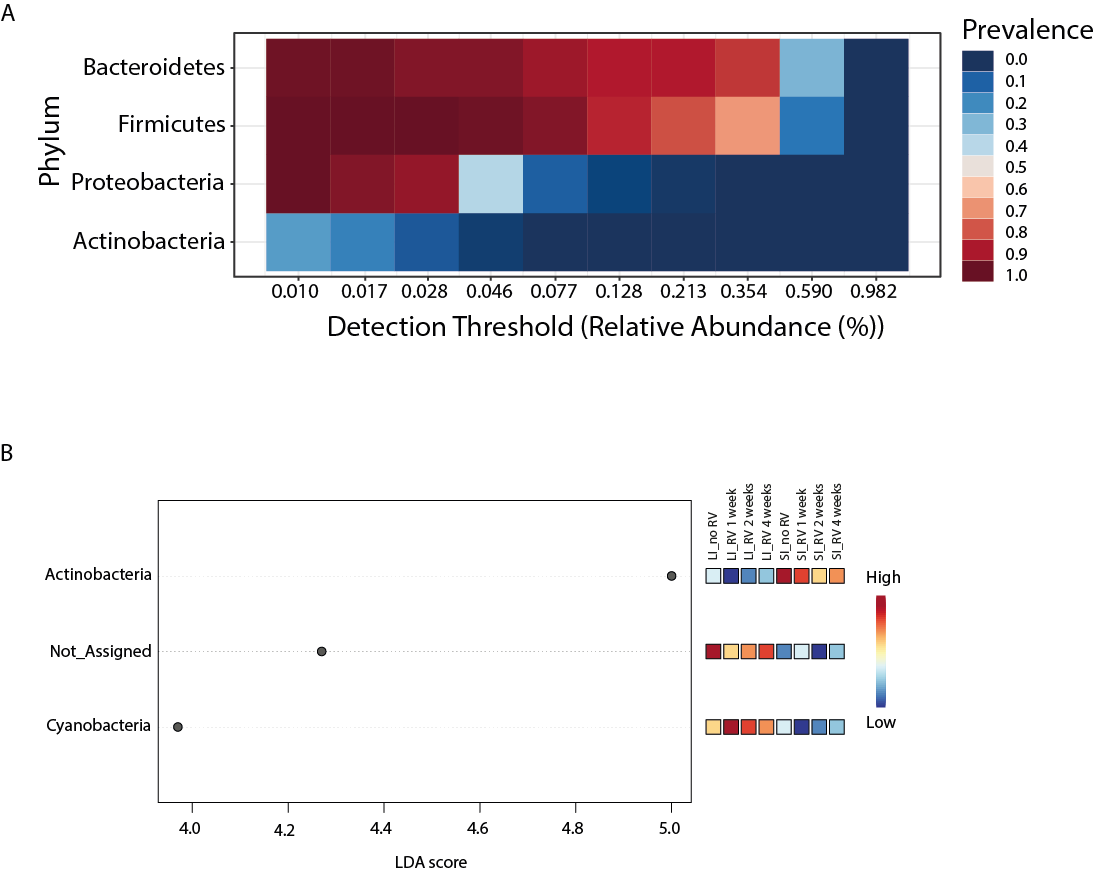
**

**Supplementary figure 3: Core microbiome detection**

(A) Heatmap representing the core microbiome at the Phylum level.

(B) Graphical summary of LEfSe analysis at Phylum level. Significant Phyla are ranked in decreasing order by their LDA scores.

Results shown are from a total of 76 intestinal samples (38 from SI and 38 from LI) from two independent experiments.


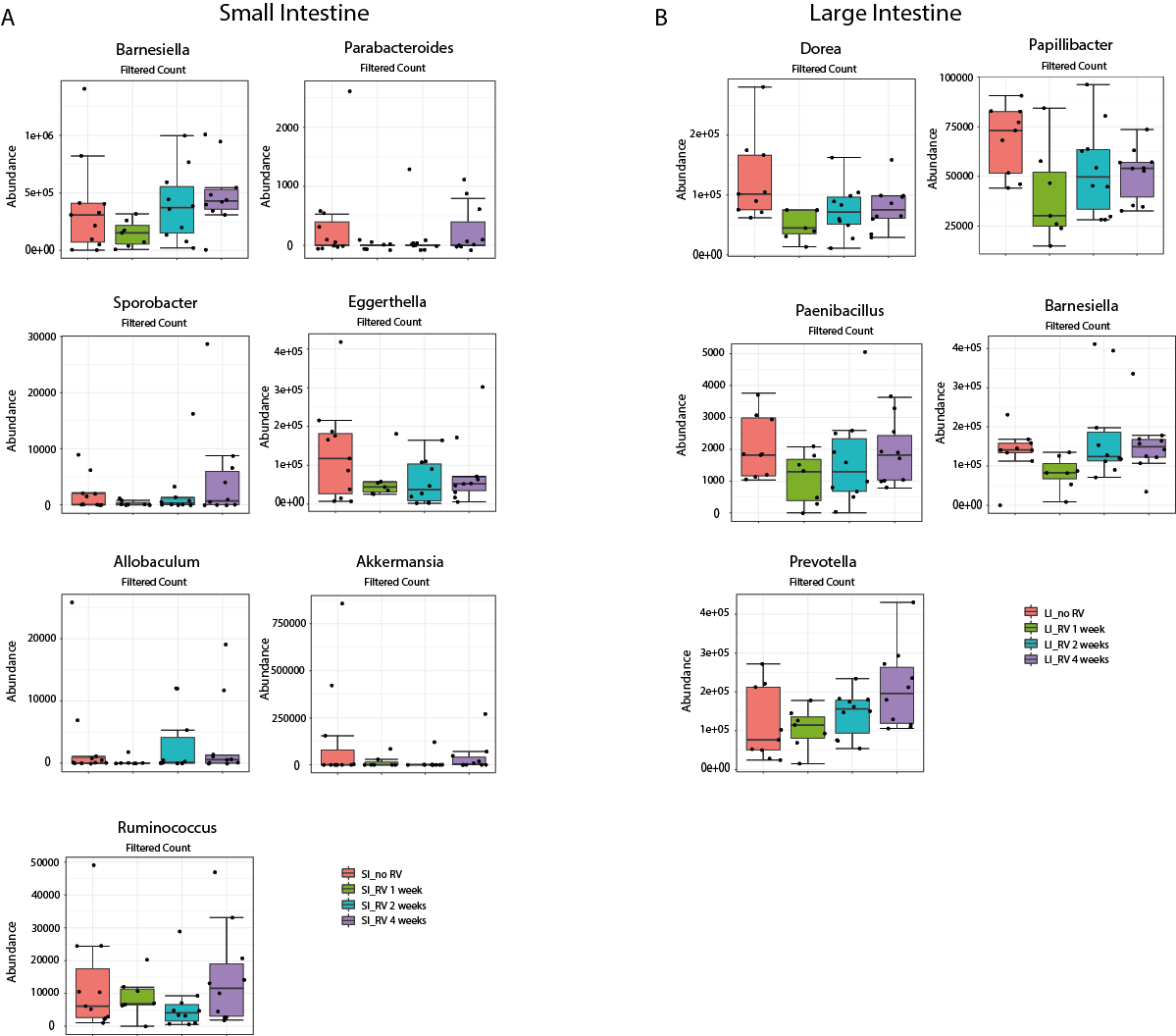


**Supplementary figure 4: Abundance of Specific Bacterial Taxa.**

(A) Abundance of specific bacterial taxa from small intestine samples of all 4 groups (no RV and 1, 2 and 4 weeks post infection).

(B) Abundance of specific bacterial taxa from large intestine samples of all 4 groups (no RV and 1, 2 and 4 weeks post infection).

Results shown are from a total of 76 intestinal samples (38 from SI and 38 from LI) from two independent experiments.


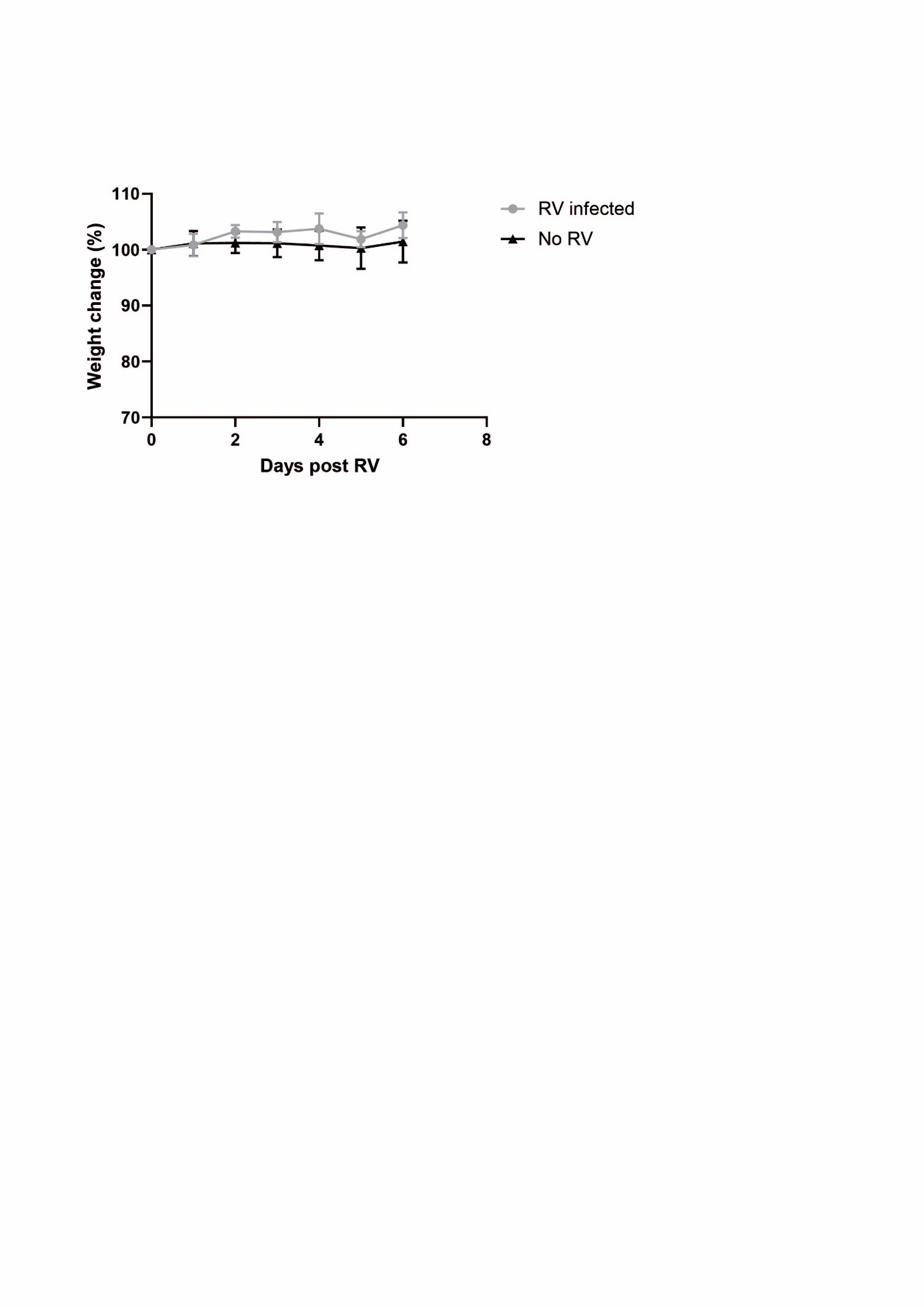


**Supplementary figure 5: Effects of RV infection on weight change.**

Weight change post RV infection from 4 RV infected and 6 non-infected mice. Results were shown as mean ± SD.


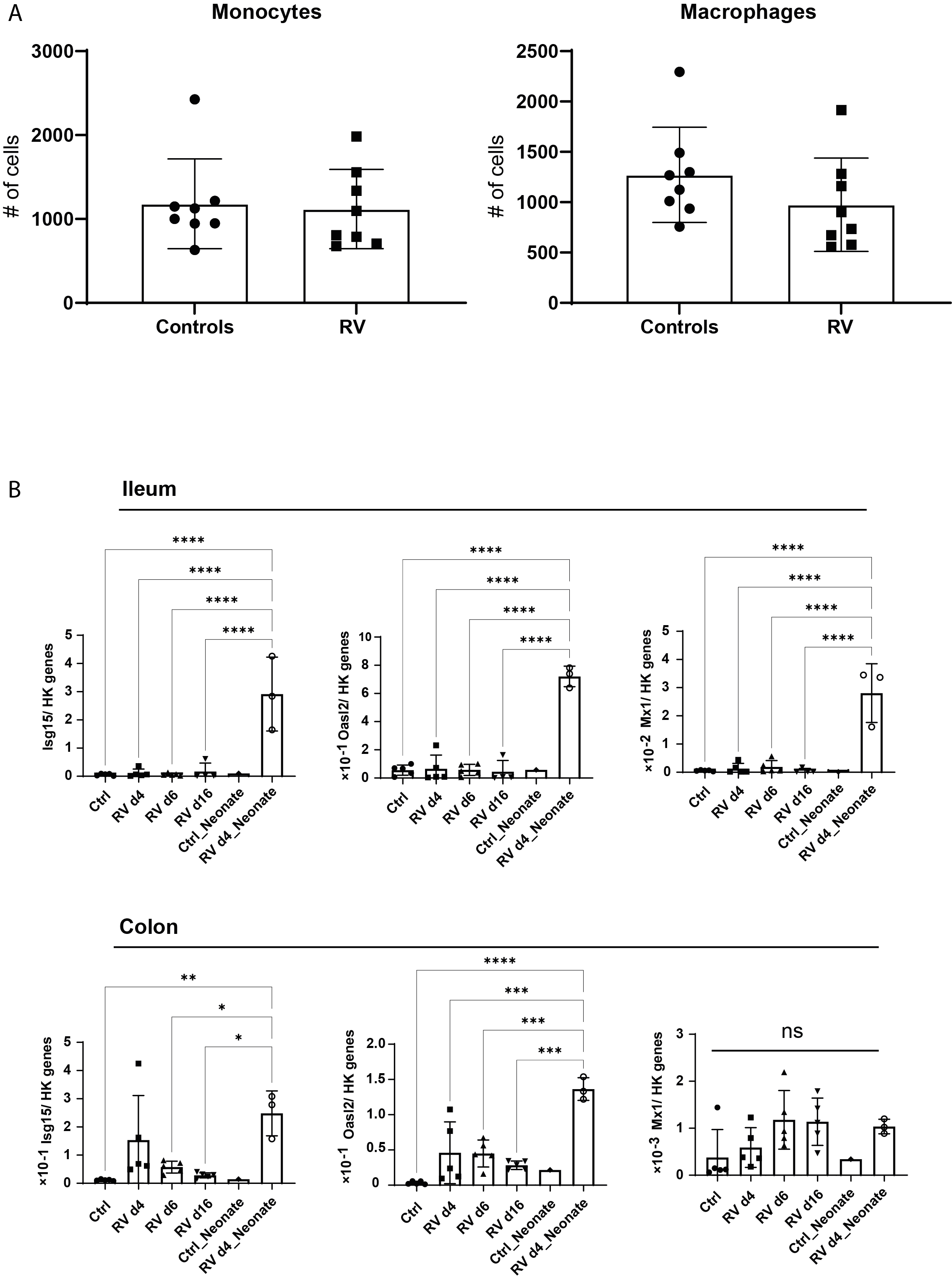


**Supplementary Figure 6: Monocyte and Macrophage abundance from colon tissue and Type I IFN gene expression from colon and Ileum tissues after RV challenge.**

(A) Mice were orally infected with RV 6 days prior to analysis. Plots depict Ly6C^+^ monocytes and Ly6C^-^MHCII^+^ macrophages from the colon, pre-gated on CD11b^+^Ly6G^-^CD64^+^CD45^+^lin^-^ live cells. Results are pooled from two independent experiments with 4 mice each.

(B) Whole tissue gene expression levels of Isg15, Oasl2 and Mx1measured by qPCR from control and orally RV-infected adult mice at day 4, day 6 and day 16 post infection. Day 4 post RV infection samples from neonate were included as a positive control. The experiment was performed once with 5 adult mice per group, 3 neonatal samples and one neonatal control sample. Results are shown as mean ± SD. (*P < 0.05, **P < 0.01, ***P < 0.001)
