# Supplementary material for: Minor alterations in the intestinal microbiota composition upon Rotavirus infection do not affect susceptibility to DSS colitis
Source: Sci Rep. 2021 Jun 29;11:13485. doi: 10.1038/s41598-021-92796-7 (PMC8242028; doi:10.1038/s41598-021-92796-7)
Supplement: Supplementary file 2 — Supplementary Tables. [file 41598_2021_92796_MOESM2_ESM.docx]

**Supplementary Tables**

Table S1A shows the important features identified by linear discriminant analysis (LDA) of effect size (LEfSe) method at the Genus level from small intestine samples.

| Genus | P values | FDR | | SI_no RV | | SI_RV  1 week | | SI_RV  2 weeks | | SI_RV  4 weeks | | LDA score |
| --- | --- | --- | --- | --- | --- | --- | --- | --- | --- | --- | --- | --- |
| Bacillus | 0.05299 | 0.72628 | 795.52 | | 1666.8 | | 26.517 | | 185.62 | | 2.91 | |
| Barnesiella | 0.06856 | 0.72628 | 368300 | | 146260 | | 397740 | | 490020 | | 5.24 | |
| Atopostipes | 0.071854 | 0.72628 | 39583 | | 0 | | 0 | | 901.59 | | 4.3 | |
| Desulfovibrio | 0.082849 | 0.72628 | 1639.3 | | 2121.4 | | 2413.1 | | 5515.6 | | 3.29 | |
| Staphylococcus | 0.10087 | 0.72628 | 13500 | | 795.52 | | 8777.3 | | 106.07 | | 3.83 | |
| Pediococcus | 0.17699 | 0.96865 | 2555.3 | | 1250.1 | | 795.52 | | 185.62 | | 3.07 | |
| Prevotella | 0.20915 | 0.96865 | 4387.4 | | 644 | | 7504.4 | | 2280.5 | | 3.54 | |
| Weissella | 0.21731 | 0.96865 | 2410.7 | | 1704.7 | | 185.62 | | 928.11 | | 3.05 | |
| Parabacteroides | 0.2572 | 0.96865 | 361.6 | | 0 | | 132.59 | | 238.66 | | 2.26 | |
| Alistipes | 0.38526 | 0.96865 | 32496 | | 17426 | | 14558 | | 26040 | | 3.95 | |
| Allobaculum | 0.39083 | 0.96865 | 3182.1 | | 265.17 | | 2996.5 | | 3420.8 | | 3.2 | |
| Ruminococcus | 0.39598 | 0.96865 | 12608 | | 8940.2 | | 6390.7 | | 14956 | | 3.63 | |
| Eggerthella | 0.40566 | 0.96865 | 130350 | | 60573 | | 57384 | | 81329 | | 4.56 | |
| Butyricicoccus | 0.43489 | 0.96865 | 3061.6 | | 1401.6 | | 7796.1 | | 1034.2 | | 3.53 | |
| Streptococcus | 0.44685 | 0.96865 | 6653.5 | | 3220 | | 742.49 | | 2943.4 | | 3.47 | |
| Sporobacter | 0.47024 | 0.96865 | 1880.3 | | 265.17 | | 2200.9 | | 4932.2 | | 3.37 | |
| Dorea | 0.50306 | 0.96865 | 83843 | | 33564 | | 57861 | | 50728 | | 4.4 | |
| Hespellia | 0.51252 | 0.96865 | 289.28 | | 151.53 | | 26.517 | | 53.035 | | 2.12 | |
| Paenibacillus | 0.51535 | 0.96865 | 265.17 | | 189.41 | | 291.69 | | 424.28 | | 2.07 | |
| Lactonifactor | 0.53814 | 0.96865 | 1880.3 | | 2576 | | 2413.1 | | 2174.4 | | 2.54 | |
| Paraeggerthella | 0.57335 | 0.98288 | 2410.7 | | 2576 | | 2280.5 | | 1962.3 | | 2.49 | |
| Papillibacter | 0.62389 | 0.98293 | 5641 | | 1856.2 | | 5250.5 | | 4746.6 | | 3.28 | |
| Not_Assigned | 0.6502 | 0.98293 | 7671900 | | 7665700 | | 6927400 | | 7351600 | | 5.57 | |
| Clostridium | 0.65528 | 0.98293 | 121020 | | 77772 | | 105730 | | 100290 | | 4.33 | |
| Defluviitalea | 0.68686 | 0.98845 | 1157.1 | | 265.17 | | 1378.9 | | 1140.3 | | 2.75 | |
| Akkermansia | 0.74122 | 0.98845 | 130680 | | 16176 | | 12357 | | 41606 | | 4.77 | |
| Pseudomonas | 0.80177 | 0.98845 | 358370 | | 137550 | | 236380 | | 215030 | | 5.04 | |
| Odoribacter | 0.8192 | 0.98845 | 13476 | | 12274 | | 3341.2 | | 3845 | | 3.7 | |
| Bacteroides | 0.82675 | 0.98845 | 5568.7 | | 5189.8 | | 9148.5 | | 5807.3 | | 3.3 | |
| Marvinbryantia | 0.85365 | 0.98845 | 2073.2 | | 1704.7 | | 1113.7 | | 1007.7 | | 2.73 | |
| Lactobacillus | 0.92005 | 0.98845 | 947250 | | 1772700 | | 2076500 | | 1559700 | | 5.75 | |
| Butyrivibrio | 0.92509 | 0.98845 | 1108.9 | | 568.23 | | 2572.2 | | 1193.3 | | 3 | |
| Olsenella | 0.93136 | 0.98845 | 988.38 | | 984.93 | | 344.73 | | 609.9 | | 2.51 | |
| Candidatus_Arthromitus | 0.95076 | 0.98845 | 22612 | | 15418 | | 25377 | | 15221 | | 3.71 | |
| Anaerorhabdus | 0.9837 | 0.98845 | 5520.5 | | 5947.5 | | 20498 | | 6947.6 | | 3.87 | |
| Lachnospira | 0.98845 | 0.98845 | 192.85 | | 303.06 | | 106.07 | | 928.11 | | 2.61 | |

Table S1B shows the important features identified by linear discriminant analysis (LDA) of effect size (LEfSe) method at the Genus level from large intestine samples.

| Genus | P values | | FDR | | LI_no RV | | LI_RV  1 week | | LI_RV  2 weeks | | LI_RV  4 weeks | | LDA score |
| --- | --- | --- | --- | --- | --- | --- | --- | --- | --- | --- | --- | --- | --- |
| Eggerthella | 0.012097 | 0.3871 | | 3225.9 | | 777.68 | | 2047.9 | | 2138.6 | | 3.09 | |
| Dorea | 0.028639 | 0.45823 | | 125290 | | 50382 | | 74242 | | 79232 | | 4.57 | |
| Prevotella | 0.069156 | 0.5746 | | 115540 | | 105890 | | 143480 | | 208240 | | 4.71 | |
| Barnesiella | 0.071825 | 0.5746 | | 136830 | | 82193 | | 179580 | | 153460 | | 4.69 | |
| Papillibacter | 0.1025 | 0.656 | | 68522 | | 40569 | | 53387 | | 51469 | | 4.15 | |
| Parabacteroides | 0.12965 | 0.66223 | | 9087.3 | | 10332 | | 23512 | | 10304 | | 3.86 | |
| Lactobacillus | 0.169 | 0.66223 | | 3153.9 | | 8165.6 | | 7530.5 | | 4964.2 | | 3.4 | |
| Ruminococcus | 0.1713 | 0.66223 | | 77926 | | 59992 | | 53647 | | 69485 | | 4.08 | |
| Marvinbryantia | 0.18669 | 0.66223 | | 17786 | | 12332 | | 5988.1 | | 8087.8 | | 3.77 | |
| Anaerorhabdus | 0.22993 | 0.66223 | | 5242.1 | | 5110.4 | | 7569.4 | | 2346 | | 3.42 | |
| Shuttleworthia | 0.23633 | 0.66223 | | 72.007 | | 518.45 | | 12.961 | | 51.845 | | 2.4 | |
| Butyricicoccus | 0.25223 | 0.66223 | | 98160 | | 58289 | | 56991 | | 48449 | | 4.4 | |
| Paenibacillus | 0.26903 | 0.66223 | | 2073.8 | | 1073.9 | | 1659 | | 1892.3 | | 2.7 | |
| Lactonifactor | 0.33224 | 0.70642 | | 7719.1 | | 42513 | | 7063.9 | | 3071.8 | | 4.29 | |
| Gemmiger | 0.35498 | 0.70642 | | 2059.4 | | 129.61 | | 362.92 | | 492.53 | | 2.98 | |
| Butyrivibrio | 0.39821 | 0.70642 | | 19499 | | 11017 | | 20958 | | 11432 | | 3.7 | |
| Alistipes | 0.41098 | 0.70642 | | 729000 | | 641340 | | 604550 | | 707100 | | 4.79 | |
| Clostridium | 0.4295 | 0.70642 | | 901570 | | 776970 | | 865800 | | 668410 | | 5.07 | |
| Akkermansia | 0.44268 | 0.70642 | | 37141 | | 7647.1 | | 12015 | | 47023 | | 4.29 | |
| Bacteroides | 0.48502 | 0.70642 | | 292290 | | 372470 | | 324910 | | 327740 | | 4.6 | |
| Acholeplasma | 0.52668 | 0.70642 | | 86.408 | | 536.97 | | 77.768 | | 362.92 | | 2.36 | |
| Defluviitalea | 0.53583 | 0.70642 | | 67053 | | 67769 | | 83665 | | 50990 | | 4.21 | |
| Pediococcus | 0.54679 | 0.70642 | | 43.204 | | 333.29 | | 298.11 | | 0 | | 2.22 | |
| Lachnospira | 0.54782 | 0.70642 | | 15712 | | 11221 | | 16513 | | 18638 | | 3.57 | |
| Hespellia | 0.56013 | 0.70642 | | 4075.6 | | 666.58 | | 907.29 | | 1918.3 | | 3.23 | |
| Roseburia | 0.57397 | 0.70642 | | 849.68 | | 1388.7 | | 2164.5 | | 3525.5 | | 3.13 | |
| Not_Assigned | 0.65026 | 0.77068 | | 6845200 | | 7222600 | | 7086700 | | 7101000 | | 5.28 | |
| Sporobacter | 0.67843 | 0.77535 | | 73663 | | 44568 | | 58948 | | 64003 | | 4.16 | |
| Odoribacter | 0.77317 | 0.85315 | | 324090 | | 341010 | | 287530 | | 336380 | | 4.43 | |
| Candidatus_Arthromitus | 0.85255 | 0.90938 | | 446.44 | | 425.87 | | 894.33 | | 557.33 | | 2.37 | |
| Desulfovibrio | 0.93005 | 0.93958 | | 15453 | | 20220 | | 15916 | | 15709 | | 3.38 | |
| Pseudomonas | 0.93958 | 0.93958 | | 1094.5 | | 1499.8 | | 1036.9 | | 1542.4 | | 2.4 | |
